# The Role of Exercise in Reducing Hyperlipidemia-Induced Neuronal Damage in Apolipoprotein E-Deficient Mice

**DOI:** 10.1155/2021/5512518

**Published:** 2021-08-06

**Authors:** Yumeng Bai, Yali Feng, Bo Jiang, Yan Yang, Zuowei Pei, Qin Yang, Yanzeng Cui

**Affiliations:** ^1^Department of Neurology, Gongyi People's Hospital, No. 117 Renmin Road, Gongyi 451200, China; ^2^Department of Neurological Rehabilitation, Gongyi People's Hospital, No. 117 Renmin Road, Gongyi 451200, China; ^3^Department of General Practice, Gongyi People's Hospital, No. 117 Renmin Road, Gongyi 451200, China; ^4^Department of Cardiology, Beijing Hospital, National Center of Gerontology, Institute of Geriatric Medicine, Chinese Academy of Medical Sciences, No. 1 Dahua Road, Beijing 100730, China; ^5^Department of Internal Medicine, Affiliated Zhongshan Hospital of Dalian University, No. 6 Jiefang Street, Dalian 116001, China

## Abstract

Hyperlipidemia causes nervous system-related diseases. Exercise training has developed into an established evidence-based treatment strategy that is beneficial for neuronal injury. This study investigated the effect of exercise on hyperlipidemia-induced neuronal injury in apolipoprotein E-deficient (ApoE^−/−^) mice. Male ApoE^−/−^ mice (age: 8 weeks) were randomly divided into four groups as follows: mice fed a normal diet (ND), normal diet+swimming training (ND+S), high-fat diet (HD), and high-fat diet+swimming (HD+S). Exercise training consisted of swimming for 40 min/day, 5 days/week for 12 weeks. After 12 weeks, we measured serum levels of total cholesterol (TC), triglyceride (TG), and low-density lipoprotein cholesterol (LDL-c). We also evaluated glial fibrillary acidic protein (GFAP) expression levels using immunohistochemistry, real-time PCR, and immunoblotting. In addition, NLR family pyrin domain-containing 3 (NLRP3), interleukin- (IL-) 18, caspase-1, Bax, Bcl-2, and phosphorylated extracellular signal-regulated kinase (p-ERK) expression levels were measured using immunoblotting. Serum levels of TG, TC, and LDL-c were lower in ApoE^−/−^ HD+S mice than in ApoE^−/−^ HD mice. Immunohistochemistry, real-time PCR, and immunoblotting showed increased levels of GFAP in the ApoE^−/−^ HD group. Immunoblotting revealed increased levels of NLRP3, IL-18, caspase-1, Bax, Bcl-2, and p-ERK in the ApoE^−/−^ HD group; however, they were significantly suppressed in the ApoE^−/−^ HD+S group. Therefore, exercise has protective effects against neuronal injury caused by hyperlipidemia.

## 1. Introduction

Hyperlipidemia is a lipid metabolism disorder that causes elevated serum total cholesterol (TC), low-density lipoprotein cholesterol (LDL-c), and triglyceride (TG) levels and/or decreased high-density lipoprotein cholesterol levels. Many studies have revealed that hyperlipidemia is a major risk factor for atherosclerosis, cardiocerebrovascular disease, chronic kidney damage, and fatty liver disease [1, 2]. There is considerable evidence that hyperlipidemia also leads to neuroinflammation, followed by neuronal damage [3, 4].

Numerous studies have reported that hyperlipidemia may cause nervous system-related diseases [5, 6]. For example, hyperlipidemia has been reported to be related to neurodegenerative diseases such as Alzheimer's and Niemann-Pick disease [7, 8]. Paul et al. also reported that hypercholesterolemia increased midbrain dopaminergic neurodegeneration in a mouse model of Parkinson's disease [9]. Hyperlipidemia is an independent risk factor for dementia [10], and high-fat diet- (HD-) induced obesity is associated with an increased risk of type 2 diabetes and impaired neural functions [11].

A review article concluded that aerobic exercise and the combination of aerobic and resistance training have effects on the serum levels of cholesterol and lipids [12]. In addition, exercise reduced neuronal injury in several studies. For example, treadmill training significantly increased the formation of lumbar spinal synapses [13]. Similarly, step training after transection of newborn rat spinal cords can increase motor neuron synapse activation [14].

Apolipoprotein E-deficient (ApoE^−/−^) mice, a well-established animal model of hyperlipidemia, have been used extensively to study the effects of atherosclerosis and renal injury [15–17]. Therefore, we established a hyperlipidemia-induced neuron injury model in ApoE^−/−^ mice by administering a HD and subjecting the mice to exercise in the form of swimming for 40 min/day, 5 days/week for 12 weeks. This study determined the effect of exercise on hyperlipidemia-induced neuronal injury in ApoE^−/−^ mice and the specific mechanisms involved.

## 2. Materials and Methods

### 2.1. Animals

Eight-week-old male ApoE^−/−^ mice were obtained from Beijing Vital River Laboratories Animal Technology Co., Ltd. (Beijing, China). All mice were given ad libitum access to food and water and were housed in a room having 40-60% humidity, at 24-26°C with a 12 h light/dark cycle. ApoE^−/−^ mice were divided randomly into four groups of 7 mice each: normal diet (ND), normal diet+exercise training (ND+S), high-fat diet (HD), and high-fat diet+swimming exercise training (HD+S). The HD mouse food comprised 1.25% (*w*/*w*) cholesterol, 22.5% (*w*/*w*) protein, 20.0% (*w*/*w*) cocoa fat, and 45.0% carbohydrate (Jiangsu Medicience, Jiangsu, China). One week prior to administration of the test diets, exercise training was initiated in an experimental swimming pool (temperature, 30°C; water depth, 44 cm; and radius, 120 cm). The progressive exercise program initially involved swimming for 5–10 min and was gradually extended to 30 min/day. When the test diets were implemented, the mice were subjected to formal swimming exercise for 40 min/day, 5 days/week for 12 weeks. All animal experiments were approved by Gongyi People's Hospital.

### 2.2. Biochemical Measurements

Blood samples were taken from the abdominal aorta of rats, and serum was stored at -80°C. TC, TG, and LDL-c levels were measured using enzyme-linked immunosorbent assay kits (Nanjing Jiancheng Bioengineering Institute, Nanjing, China) according to the manufacturer's protocols. The TC, TG, and LDL-c concentrations were calculated based on measurements of optical density at the respective wavelengths for each compound according to the manufacturer's protocol.

### 2.3. Immunohistochemistry

The brains of all mice were perfusion-fixed with 4% paraformaldehyde in 0.1 M sodium phosphate buffer (pH 7.4) following a heparinized saline flush. The brains were dehydrated and embedded in paraffin. Serial 7 *μ*m coronal sections were cut using a microtome. Paraffin sections of the hippocampus were used for the immunohistochemical analysis, which was performed using the Histone Simple stain kit (Nichirei, Tokyo, Japan) according to the manufacturer's instructions. Paraffin-embedded sections were deparaffinized with xylene and then rehydrated in a descending series of ethanol washes. The sections were treated for 15 min with 3% H_2_O_2_ in methanol to inactivate endogenous peroxidases and then incubated at 4°C overnight with a primary antibody against glial fibrillary acidic protein (GFAP; rabbit anti-GFAP, 1 : 500; Z0334, Dako, Carpinteria, CA, USA). All sections were examined microscopically using a BX40 upright light microscope (Olympus, Tokyo, Japan).

### 2.4. Western Blotting

Mice in each group were euthanized by intraperitoneal injection of an overdose of sodium pentobarbital. The hippocampus and cortex were isolated from each brain (*n* = 5 in each group). Briefly, the hippocampus was homogenized (1 : 5, *w* : *v*) in ice-cold lysis buffer containing 50 mM Tris-HCl (pH 7.4), 150 mM NaCl, 1% Nonidet P-40, 1 mM EDTA, 0.25% sodium deoxycholate, 0.1% sodium dodecyl sulfate, protease inhibitor cocktail, and phosphatase inhibitor cocktail (1 : 100 each; Nacalai Tesque, Kyoto, Japan). The resulting homogenates were centrifuged at 12,000 × *g* for 30 min at 4°C, the supernatants were collected, and total protein levels were determined using a bicinchoninic acid assay kit (Pierce, Rockford, IL, USA). Proteins (15 *μ*g) were separated on 12% sodium dodecyl sulfate-polyacrylamide gels and transferred onto polyvinylidene fluoride membranes in a wet transfer device (30 V, 1 h). Membranes were preincubated in 5% bovine serum albumin for 2 h and then incubated with the following primary antibodies overnight at 4°C: rabbit anti-GFAP, anti-NLRP3, anti-caspase-1, anti-interleukin- (IL-) 18, anti-Bax, anti-Bcl2, and anti-p-ERK (all at 1 : 1000 dilution and from ProteinTech Group, Rosemont, IL, USA). After incubation with horseradish peroxidase-conjugated anti-rabbit secondary antibodies, as appropriate, for 1 h (1 : 5000; KPL, Gaithersburg, MD, USA), the membranes were reacted with an enhanced chemiluminescence reagent (New England Lab, Woburn, MA, USA). Finally, specific protein bands were visualized by using the ImageQuant LAS 4000 imaging system (GE Healthcare Life Sciences, Issaquah, WA, USA). Intensities of the protein bands were quantified using ImageJ software (NIH; https://imagej.nih.gov/ij/).

### 2.5. RNA Isolation and Real-Time RT-PCR

Total DNA was isolated from the cerebral cortex and hippocampus tissues. Then, according to the manufacturer's protocol, TransScript One-Step gDNA Removal and cDNA Synthesis SuperMix kits (TransGen, Beijing, China) were used to prepare complementary DNA. The GFAP gene expressions were analyzed in terms of quantity by running RT-PCR by the use of fluorescent SYBR Green technology. Relative expression of the GFAP gene was normalized to *β*-actin. The primer was as follows: *β*-actin: forward primer: 5-CGATGCCCTGAGGGTCTTT-3′ and reverse primer: 5′-TGGATGCCACAGGATTCCAT-3′, and GFAP: forward primer: 5′-TTGCTGGAGGGCGAAGAAA-3′ and reverse primer: 5′-AGGGAGAGCTGGCAGG-3′.

### 2.6. Statistical Analyses

Data are presented as the mean ± standard error of mean and were analyzed using SPSS software version 23.0 (IBM, Chicago, IL, USA). Intergroup differences were determined by an analysis of variance and Tukey's post hoc test. *P* < 0.05 was regarded as significant.

## 3. Results

### 3.1. Metabolic Characterization

**Figure 1:**
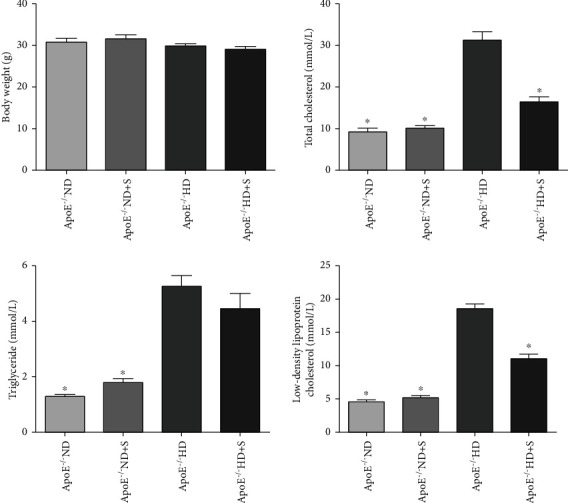
Body weights and total cholesterol, triglyceride, and low-density lipoprotein levels in mice after 12 weeks of feeding with different diets. Data are presented as the mean ± standard error of the mean. *n* = 7 per group. ApoE^−/−^: apolipoprotein E-deficient; HD: high-fat diet; ND: normal diet; S: swimming.

The metabolic characteristics of the animals are shown in Figure 1. Body weights did not differ significantly among the four groups. The levels of LDL-c, TC, and TGs were significantly increased (*P* < 0.05) in the ApoE^−/−^ HD groups compared with the ND and ND+S groups. In addition, the levels of LDL-c and TC were decreased in the HD+S group compared to the HD group. However, TG levels did not show this decrease. These results suggest that exercise was effective in reducing TC and LDL levels in HD mice.

### 3.2. Exercise Inhibits the Increased GFAP Expression Caused by Hyperlipidemia in the Cerebral Cortical Layer and Hippocampal Area

**Figure 2:**
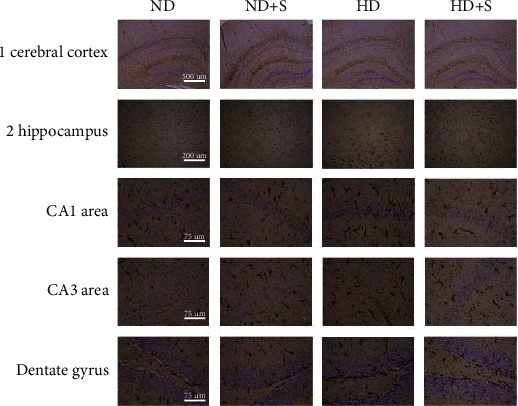
Immunohistochemistry showing glial fibrillary acidic protein (GFAP) expression in the (1) cerebral cortex and (2) hippocampus CA1 area, hippocampus CA3 area, and dentate gyrus. ApoE^−/−^: apolipoprotein E-deficient; HD: high-fat diet; ND: normal diet; S: swimming.

**Figure 3:**
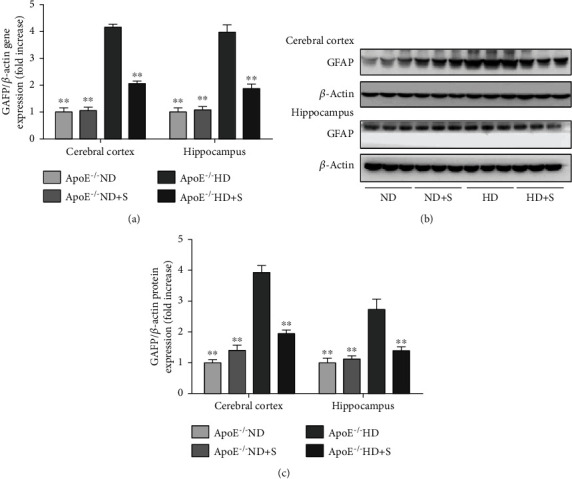
(a) Relative mRNA expression of glial fibrillary acidic protein (GFAP) in the cerebral cortex and hippocampus area. (b) Immunoblotting for GFAP protein expression in the cerebral cortex and hippocampus area. Bar graphs showing quantification of GFAP protein expression. Data are presented as the mean ± standard error of mean; *n* = 3 per group. ^∗∗^*P* < 0.01 vs. the ApoE^−/−^ HD group. ^∗^*P* < 0.05 vs. the ApoE^−/−^ HD group. ApoE^−/−^: apolipoprotein E-deficient; HD: high-fat diet; ND: normal diet; S: swimming.

To investigate the pathological changes in hyperlipidemia-induced neuroinflammation, we performed GFAP immunohistochemical staining (Figure 2) of different brain tissues (cerebral cortex, hippocampal CA1 area, hippocampal CA3 area, and dentate gyrus). The results showed that GFAP-positive cells were increased in the ApoE^−/−^ HD group compared with the ND and ND+S groups. Interestingly, swimming exercise (HD+S group) decreased the number of GFAP-positive cells. In addition, in the ND group, GFAP-positive glial cells had small nuclei and short protrusions, while in the HD group, glial cells were activated, the nuclei were enlarged and rounded, and the protrusions were elongated. After exercise, glial cell activity decreased, the nuclei became smaller, and the protrusions shrank compared to the HD group. Furthermore, real-time PCR and western blot analysis of GFAP showed that the expression of GFAP was inhibited by exercise (Figure 3).

### 3.3. Exercise Inhibits Hyperlipidemia-Induced Neuroinflammation

**Figure 4:**
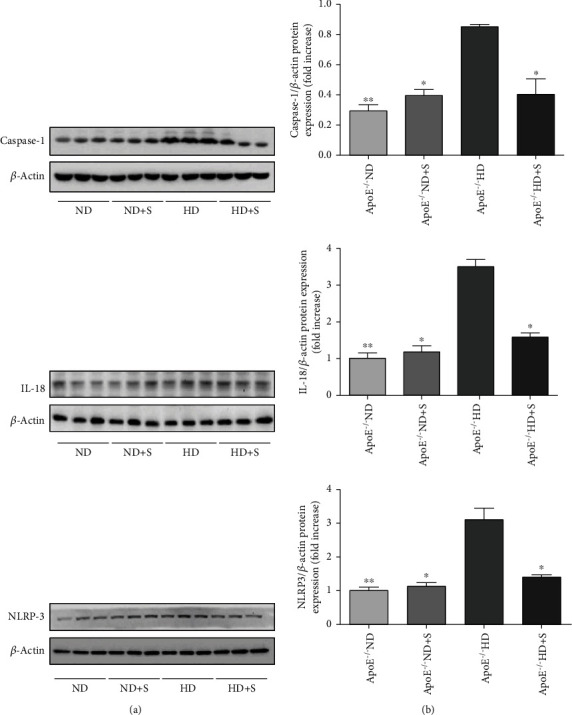
(a) Immunoblotting for NLR family pyrin domain-containing 3 (NLRP3), interleukin- (IL-) 18, and caspase-1 protein expression in neuronal tissues. (b) Bar graphs showing quantification of NLRP3, IL-18, and caspase-1 protein expression. Data are presented as the mean ± standard error of mean; *n* = 3 per group. ^∗∗^*P* < 0.01 vs. the ApoE^−/−^ HD group. ^∗^*P* < 0.05 vs. the ApoE^−/−^ HD group. ApoE^−/−^: apolipoprotein E-deficient; HD: high-fat diet; ND: normal diet; S: swimming.

To evaluate the involvement of proinflammatory cytokines and cell death factors in neuronal tissues from each of the four groups, NLRP3, IL-18, and caspase-1 protein expression were measured using western blotting (Figure 4). All three proteins were increased in the HD group compared to the ND group. However, these increases were attenuated in the HD+S group.

### 3.4. Exercise Inhibits Apoptosis Induced by Hyperlipidemia

**Figure 5:**
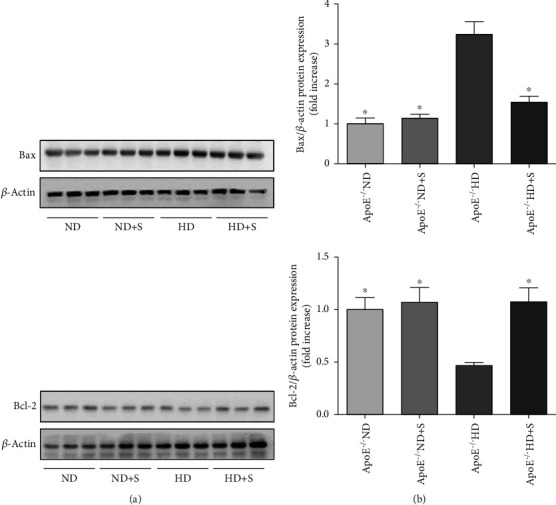
(a) Immunoblotting for Bax and Bcl-2 protein expression in neuronal tissues. (b) Bar graphs showing quantification of Bax and Bcl-2 protein expression. Data are presented as the mean ± standard error of mean; *n* = 7 per group. ^∗^*P* < 0.05 vs. the ApoE^−/−^ HD group. ApoE^−/−^: apolipoprotein E-deficient; HD: high-fat diet; ND: normal diet; S: swimming.

To evaluate apoptosis in neuronal tissues of the four experimental groups, Bax and Bcl-2 protein expression was measured using western blotting (Figure 5). The Bax protein level was higher in the HD group than in the ND group. This increase was attenuated in the HD+S group. Interestingly, the expression of Bcl-2 showed the opposite trend. Compared to the ND group, Bcl-2 expression was decreased, and this decrease was attenuated by exercise in the HD+S group.

### 3.5. p-ERK Signaling Pathway

**Figure 6:**
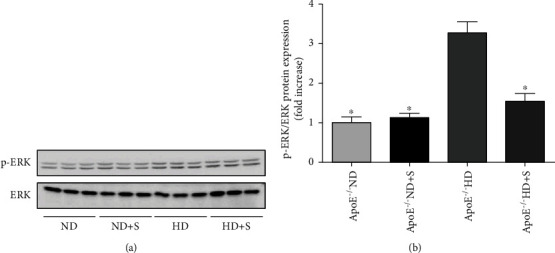
(a) Immunoblotting for phosphorylated extracellular signal-regulated kinase (p-ERK) protein expression in neuronal tissues. (b) Bar graph showing quantification of p-ERK protein expression. Data are presented as the mean ± standard error of mean; *n* = 3 per group. ^∗^*P* < 0.05 vs. the ApoE^−/−^ HD group. ApoE^−/−^: apolipoprotein E-deficient; HD: high-fat diet; ND: normal diet; S: swimming.

Western blotting analysis of p-ERK was used to investigate neuronal damage caused by hyperlipidemia (Figure 6). p-ERK protein expression in neuronal tissues was lower in ApoE^−/−^ HD+S mice than in ApoE^−/−^ HD mice.

## 4. Discussion

In this study, we explored the protective effects of exercise using a model of hyperlipidemia-induced neuronal injury in ApoE^−/−^ mice. The results demonstrated that exercise had a protective effect on this injury by affecting proinflammatory cytokine levels, apoptosis, GFAP expression, and the p-ERK pathway.

No significant variation was observed in body weights among the four groups of mice. However, compared with ApoE^−/−^ ND group mice, higher LDL-c and TC levels were observed in ApoE^−/−^ HD group mice. This result indicated that the hyperlipidemia mouse model was established successfully by a high-fat diet. A review article noted that exercise has an effect on the serum levels of cholesterol and lipids [18]. Interestingly, LDL-c and TC levels were significantly lower in the ApoE^−/−^ HD+S group than in the ApoE^−/−^ HD group, suggesting that exercise had a protective effect against lipid deposition caused by a HD. However, the effect of exercise on hyperlipidemia-induced neuronal injury remained unclear.

Immunohistochemistry, real-time PCR, and immunoblotting were performed to evaluate GFAP levels for understanding the mechanism of neuronal damage caused by hyperlipidemia. Astrocytes play a significant role in maintaining the physiological functions of the blood-brain barrier and in regulating the metabolism of lipids in the brain [19]. GFAP, an intermediate filament protein that is primarily expressed in astrocytes, is a key marker of mature astrocytes [20]. Many studies have shown that overexpression of GFAP is associated with neuronal damage. Stolmeier et al. reported that increases in GFAP and Ibal antagonized hippocampal function [21]. In the current study, the expression of GFAP was increased in the HD group. This showed that hyperlipidemia activated glial cells and caused neuronal damage. Importantly, this effect was suppressed by exercise.

Hyperlipidemia-induced inflammation plays a crucial role in the development of cardiac damage, ischemic stroke, and brain injury [22]. Neuroinflammation has been identified as a causative factor of multiple neurological diseases [23, 24]. The nucleotide-binding oligomerization domain-, leucine-rich repeat-, and pyrin domain-containing 3 (NLRP3) inflammasome is abundantly expressed in the central nervous system and is the most studied inflammasome in this system [25, 26]. Activating the NLRP3 inflammasome leads to the activation of caspase-1. Subsequently, activated caspase-1 causes the production of IL-1*β* and IL-18, as well as proinflammatory cytokines, and mediates rapid cell death. IL-1*β* and IL-18 drive inflammatory responses through a variety of downstream signaling pathways that result in neuronal injury [12, 27, 28]. Results of the current study showed that NLRP3, caspase-1, and IL-18 levels were increased in the HD group. In this study, swimming, as a treatment, reduced the expression of NLRP3, caspase-1, and IL-18, thereby indicating that exercise can act as a protective agent against hyperlipidemia-induced neuronal injury.

Apoptosis plays an important role in various neuronal injuries [29–31]. Kim et al. found that berberine treatment inhibited brain inflammation in poloxamer 407-treated hyperlipidemic rats by inhibiting apoptosis [32]. The key regulators of apoptosis are members of the Bcl-2 family of proteins; this protein family contains a variety of proapoptotic (such as Bax and Bak) and antiapoptotic (such as Bcl-2, Bcl-xL, and Bcl-w) proteins [33, 34]. In the current study, compared to the ND group, Bax protein levels were increased and Bcl-2 protein levels decreased in the HD group. It is worth noting that exercise inhibited the expression of Bax and enhanced the expression of Bcl-2, suggesting that apoptosis was inhibited.

The mitogen-activated protein kinase/ERK pathway participates in every stage of cell growth and development, including cell proliferation, differentiation, migration, senescence, and apoptosis [35]. In white matter-lesioned rats, activation of the mitogen-activated protein kinase/ERK pathway promotes neuronal apoptosis, thereby worsening the condition [36]. In the current study, we found that p-ERK expression was significantly increased in the HD group. This showed that hyperlipidemia caused neuronal injury by activating the p-ERK signaling pathway, and exercise can inhibit this effect.

## 5. Conclusions

In conclusion, the results of the present study showed that exercise treatment had a protective effect on hyperlipidemia-induced neuronal injury in ApoE^−/−^ mice. Exercise reduced the increases in serum LDL-c and TC and protected against neuronal damage by inhibiting GFAP expression, inflammation, apoptosis, and activation of the p-ERK signaling pathway. The findings of this study could be beneficial in developing novel strategies for the prevention and treatment of neuronal injury.

## Data Availability

All datasets are available from the corresponding author upon reasonable request.
